# Stem cells of fallopian tube mucosa lost their stemness characteristics under prolonged conditions

**DOI:** 10.5935/1518-0557.20210097

**Published:** 2022

**Authors:** Somasundaram Indumathi, Marappagounder Dhanasekaran, Kaingade Pankaj, Ganneru Sireesha, Badodekar Nilaja, Vyas Nishant, Bhonde Ramesh

**Affiliations:** 1 Department of Stem cells and Regenerative Medicine, D. Y. Patil University, Kolhapur, Maharashtra, India; 2 Laboratory Director, Reelabs Pvt. Ltd., Mumbai, Maharashtra, India; 3 Laboratory Director & Consultant Embryologist, ReproHelix Labs, Kolhapur, Maharashtra, India; 4 Laboratory Director & Consultant Embryologist, Sunanda IVF & Fertility Clinic, Kolhapur, Maharashtra, India; 5 Department of Biochemistry, Acharya Nagarjuna University, Guntur, Andhra Pradesh, India; 6 Research Associate, Logical Life Sciences Pvt. Ltd. Pune, Maharashtra, India; 7 Research Head, Logical Life Sciences Pvt. Ltd. Pune, Maharashtra, India; 8 Director Research, D.Y. Patil Vidyapeet, Pimpri-Pune, Maharashtra, India

**Keywords:** fallopian tube mucosa, mesenchymal stem cells, proliferation, multipotent differentiation, long-term culture

## Abstract

**Objective:**

Stem cells have been identified from various adult sources, including bone marrow, adipose tissue, and placenta, to name a few. Recently, the fallopian tube has also been identified as a novel source of therapeutics. However, the ability of stem cells from the fallopian tube mucosa to retain prolonged efficacy of proliferation and differentiation is yet to be explored. This forms the basis of the present study.

**Methods:**

Stem cells isolated from the fallopian tube mucosa were tested for their marker characterization (markers of mesenchymal, pericyte, epithelial, and cell adhesion molecules) at various passages (P1, P3, P5, P10, P15). Proliferation, differentiation (osteoblast and adipocytes), and karyotyping were also carried out at both early (P3) and late (P15) passages.

**Results:**

Fallopian tube mucosa possesses mesenchymal stem cells, but they do not retain the ability to proliferate and differentiate beyond P15.

**Conclusions:**

Although fallopian tube mucosal MSCs (FT-MMSCs) possess stem cell attributes, they cannot outweigh or be used in parallel to existing stem cell sources due to their inability to retain stemness characteristics beyond P15. Since FT-MMSC studies are in their infancy, further in-depth research is warranted to test whether FT-MMSCs have a use in bench to bedside applications. FT-MMSCs might be linked to tubal inflammation and fallopian tube hyperplasia, which contributes to a possible role in diagnostics and in providing insights for the betterment of womankind.

## INTRODUCTION

Bone marrow has been the most conventional source of stem cells in regenerative medicine (Deda *et al*., 2008; Prasad *et al*., 2012; Simari *et al*., 2014; Zakrzewski *et al*., 2019). However, there are limitations in the use of bone marrow with its seemingly low number of mesenchymal stem cells (MSC), poorer yield of nucleated cells coupled with the decline in MSC volume with advancing age, variable body mass index and tissue harvest site (Pittenger *et al*., 1999; Pountos *et al*., 2007; Stolzing *et al*., 2008; Rebelatto *et al*., 2008). These features have led scientists to explore the use of stem cells of redundant tissue sources, such as adipose tissue, for therapeutic use (Parker & Katz, 2006; Locke *et al*., 2009; Zhu *et al.*, 2008; Zakrzewski *et al*., 2019). Besides, stem cells from female reproductive sources, such as the endometrium and fallopian tube, have gained equal interest in the last decade (Chan *et al*., 2004; Gargett *et al.*, 2007; 2008; Gargett & Masuda, 2010; Dimitrov *et al*., 2008; Snegovskikh *et al*., 2014; Wang *et al*., 2015; Zakrzewski *et al*., 2019). Endometrial stem cells have been widely explored in recent years (Verdi *et al*., 2014; Ghobadi *et al*., 2015; Gargett *et al*., 2016; Indumathi *et al*., 2013; Kaingade *et al*., 2017). However, stem cells of fallopian tubes (hFTs), especially from the mucosa, although sharing the same embryologic origin as the uterus, have not been characterized extensively for their *in-vitro* attributes and functionality. Fallopian tube has the capacity to undergo dynamic endocrine-induced change during the menstrual cycle, including cell growth and regeneration (Snegovskikh *et al*., 2014; Wang *et al*., 2015). It is well documented that fallopian tube-derived cells may contribute to provide the unique environment required for fertilization, early development, and implantation onto the uterus, all of which conducive to successful reproductive outcomes (Henriksen *et al*., 1990; Lyons *et al*., 2006; Li & Winuthayanon, 2017). With these attributes, tissue fragments of human fallopian tube, which are usually discarded after surgery, may represent a potential source for regenerative medicine. Although the epithelial components of the fallopian tube are well studied (Henriksen *et al*., 1990; Djahanbakhch *et al*., 1999; Karst & Drapkin, 2012), study of their mesenchymal component is just developing in recent years in both FT-MSCs and FT-MMSCs (Jazedje *et al*., 2009; Wang *et al*., 2015). However, its wide marker characterization and retention beyond P15 on FT-MMSCs is yet to be determined.

The present study looked into whether FT-MMSCs might retain their stemness characteristics under extensive *in-vitro* culture and characterization.

## MATERIAL AND METHODS

### Sampling

The study was reviewed and approved by the members of the Institutional Ethics Committee. The samples were collected after written informed consent was obtained from the patients. Human fallopian tube biopsy specimens were collected from benign, reproductively active women (n=10) with a mean age of 39.8±2 and a body mass index of 25.4±0.766 who were undergoing tubectomy. All samples were transported to the laboratory in a sterile container. The patients had not undergone exogenous hormonal treatment for at least 3 months prior to surgery.

### Isolation and culturing of MSCs

The fallopian tube samples were processed based on our previously published paper (Indumathi *et al*., 2013). Briefly, the tissues were rinsed with PBS (Cat. No: TL1006, Sigma-Aldrich) supplemented with antibiotics (Cat. No: 15240-062, Invitrogen) and incubated with 0.25% trypsin (Cat. No: T4049, Sigma-Aldrich) at 37°C for 15 minutes. Later, DMEM (Cat. No: 11885084, Invitrogen) supplemented with 10% FBS (Cat. No:10270-106, Invitrogen) was added to neutralize the enzyme. Single cell suspension was further enumerated and evaluated for its cell viability using Trypan Blue staining (Cat. No: T8154, Sigma-Aldrich). 

All obtained cells were plated at a density of 3.0×10^4^/cm^2^ in T-25 flasks (Cat. No: 156367, Nunc) and cultured in DMEM supplemented with 10% FBS and 1% antibiotic-antimycotic solution. Standard culture conditions of 37°C, 5% CO_2_ and 95% humidity were maintained until 70-80% confluence was obtained. The cells were trypsinized and subsequently passaged upon confluence.

### Flow cytometry analysis

The stem cells were examined for marker profiling on a flow cytometer (BD FACSAria II). This was carried out similar to our previously published protocol (Indumathi *et al*., 2013). Hematopoietic markers (CD34, HLA-DR, CD14), mesenchymal stem cell markers (CD73, CD105 and CD90), cell adhesion molecules (CD49d, CD166, CD106, CD54, CD29 and CD31), perivascular markers (CD140b and CD146), and other unique markers (ABCG2 and CD117) were used for characterization. Markers and their respective fluorochromes are specified in Table 1. They were analyzed using a Becton-Dickinson FACS Aria instrument. The data was acquired from 10,000 events using the FACS Diva software.

**Table 1.** Assortment of cell surface markers and their respective fluorochromes.

S.No.	MARKERS	FLUROCHROME
**1**	CD29	PE (BD Bioscience)
**2**	CD166	PE (BD Bioscience)
**3**	CD106	FITC (BD Bioscience)
**4**	CD49d	PE (BD Bioscience)
**5**	CD31	FITC (BD Bioscience)
**6**	CD54	PERCP (BD Biosciences)
**7**	CD34	PE (BD Bioscience)
**8**	CD140b	PE (BD Bioscience)
**9**	CD117	APC (e-Biosciences)
**10**	CD90	PERCP (e-Biosciences)
**11**	CD105	APC (e-Biosciences)
**12**	CD73	PE (BD Bioscience)
**13**	ABCG2	PE (e- Bioscience)
**14**	CD14	APC (e-Biosciences)
**15**	HLADR	FITC (BD Bioscience)

### Intracellular staining by flow cytometry

The cells were mixed with 500 µl of cytofix/cytoperm (Cat. No: 554722, BD Bioscience) solution to form a single cell suspension and incubated at 4°C for 20 minutes. The fixed cells were then washed with 1X wash buffer (Cat. No: 554723, BD Bioscience) at 200 g for 7 minutes. The supernatant was discarded and the cells were then resuspended with 500 µl of perm wash buffer. 1X10^6^ cells were then treated with appropriate concentrations of antibodies. All tubes were vortexed well and incubated for 45 minutes at 18°-24°C in the dark. The cells were then washed twice with perm wash buffer at 200 g for 7 minutes. The pellet was then resuspended for final flow cytometry analysis.

### Growth curve

The growth curve analysis was carried out similar to our previous paper (Dhanasekaran *et al*., 2013). In short, the rate of growth of each cell population was calculated every day for nearly 2 weeks by counting the total number of cells in duplicates. The results were allotted on a log-linear scale. The growth curve characteristics were carried out in a 12-well plate in duplicates with a seeding density of 300,000 cells per plate at day 0. The population doubling time was calculated using the formula:


$$\mathrm{PDT}=\frac{\mathrm{Days}\;\mathrm{of}\;\exp\mathrm{onential}\;\mathrm{phase}}{\left(\log\;N2-\;\log\;N1\right)/\log\;2}$$

Where, N1 is the number of cells at the beginning of the exponential growth phase and N2 is the number of cells at the end of the exponential growth phase.

### Differentiation

Differentiation studies were performed according to the previously published paper (Dhanasekaran *et al*., 2013). However, a brief protocol of each differentiation is explained below.

### Osteogenic differentiation

Upon reaching 80-90% confluence, the complete cell culture media was replaced with osteogenic induction medium with timely replacements. Osteogenic induction media consisted of DMEM-LG, 10% FBS, 1% Antibiotic, Dexamethasone (0.1 µM) (Cat. No: D4902, Sigma), β-glycerophosphate (10 mM) (Cat. No: G-9422, Sigma) and Ascorbic acid (2 mM) (Cat. No: A4544, Sigma). Osteocytes were confirmed after 21 days of differentiation by Alizarin Red and Von Kossa staining. Stained cells were visualized using a phase contrast microscope.

### Adipogenic differentiation

Similarly, adipogenic differentiation was promoted by differentiating the cells under the presence of adipogenic induction media containing DMEM-LG, 10% FBS, 1% Antibiotic, Dexamethasone (1µM), Isobutyl methyl xanthine (0.5 mM) (Cat. No: 15879, Sigma-Aldrich), Insulin (10 µg) (Cat. No: TCL035, HIMEDIA) and Indomethacin (200 µM) (Cat. No: 17378, Sigma-Aldrich). Adipogenic differentiation was assessed on the eighteenth day of culture by staining the MSC monolayer with triglyceride specific dye, Oil-O-Red.

### Staining

#### Von Kossa staining

Von Kossa staining of calcium-phosphate deposits was performed according to the manufacturer's instruction. The cells were washed 3 times with PBS before fixation with 10% formalin (Cat. No: HT50-1-2, Sigma-Aldrich) for 1 hour at room temperature. The cells were then washed 5 times with distilled water. Next, 3 ml of 2.5% silver nitrate (Cat. No: 209139, Sigma-Aldrich) were added and the cells were exposed to UV light for 45 minutes. After washing, 5% Sodium thiosulfate) (Cat. No: S0764, Sigma-Aldrich) was added for 2 minutes and the cells were again washed before nuclear fast red (Cat. No: N3020, Sigma-Aldrich) staining (Dhanasekaran *et al*., 2013).

#### Alizarin red staining

Alizarin Red Staining identifies osteoblast differentiation by the presence of calcium deposits in cells with red dye. In short, media was removed from the culture plate and fixation with 70% alcohol was performed after the cells were washed with PBS. The cells were then incubated for 1 hour in room temperature. After 1 hour, the cells were treated with 5 ml of alizarin red working solution made form stock (Cat. No: A5533, Sigma-Aldrich) and incubated at room temperature for 30 minutes. The plates were washed twice with de-ionized water and viewed under a microscope (Dhanasekaran *et al*., 2013).

#### Oil O Red staining

Adipogenic differentiation was confirmed using Oil Red O staining (Cat. No: O0625, Sigma) by quantifying lipid vacuoles. The culture medium was aspirated, the cells washed with PBS and fixed with 5 ml of 10% of formalin for 10 minutes at room temperature. The solution was replaced with 5 ml of fresh formalin and incubated for 1 hour at room temperature. The cells were washed with 5 ml of ddH2O followed by 5 ml of 60% isopropanol. The cells were completely dried and incubated with 3 ml of Oil Red O working solution for 10 minutes. The cells were washed with ddH2O and imaged under microscope (Dhanasekaran *et al*., 2013).

### Karyotyping

Karyotyping was performed at both early and late passages to verify the maintenance of chromosomal normality according to our previous publication (Dhanasekaran *et al*., 2013). Standard Giemsa staining procedure was carried out; chromosome preparations were obtained from 70 to 80% confluent cells. The cells were treated with Colcemid solution (KaryoMax-Colcemid, Invitrogen) to stop microtubule formation. The mitotically arrested cells were then harvested using 0.25% trypsin-EDTA. The extracted cells were then immersed in 75 mmol/l KCl for 30 min at room temperature and were centrifuged. The supernatant was replaced with fixative (methanol: acetic acid, 3:1) and the suspension was spread over slides for observation and imaging. At least 20 metaphase spreads were analyzed. The karyotypes were imaged using a Nikon-Eclipse-90i microscope (Nikon) using cytovision software.

### Statistical Analysis

All marker analysis from early (P3) and late passage (P15) of FTM-MSCs (n=10) were shown in Mean ± SEM. The data were analyzed by using Student's t-test and p-values were calculated to determine the statistically significant variations between P3 and P15. Results were considered statistically significant when *p*<0.05*.

## RESULTS

### Culturing of Stem Cells

**Figure 1:**
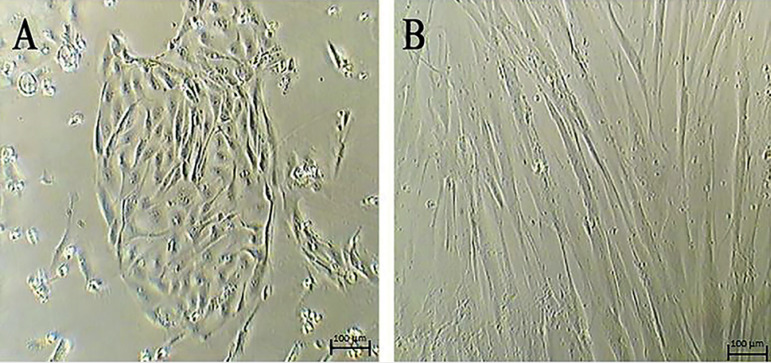
*In-vitro* culture of FT-MSCs. FT-MMSCs cultures at (a) Early passage (P3), (b) Late passage (P15); Magnification 20X.

The stem cells from human fallopian tube samples were characterized for extensive *in-vitro* culturing. FT-MSCs do not retain their fibroblast-like spindle shaped morphology at later passages with elongated fibroblastic morphology (Fig. 1). Besides, cells do not grow well beyond P15.

### Cell characterization

**Graph 1:**
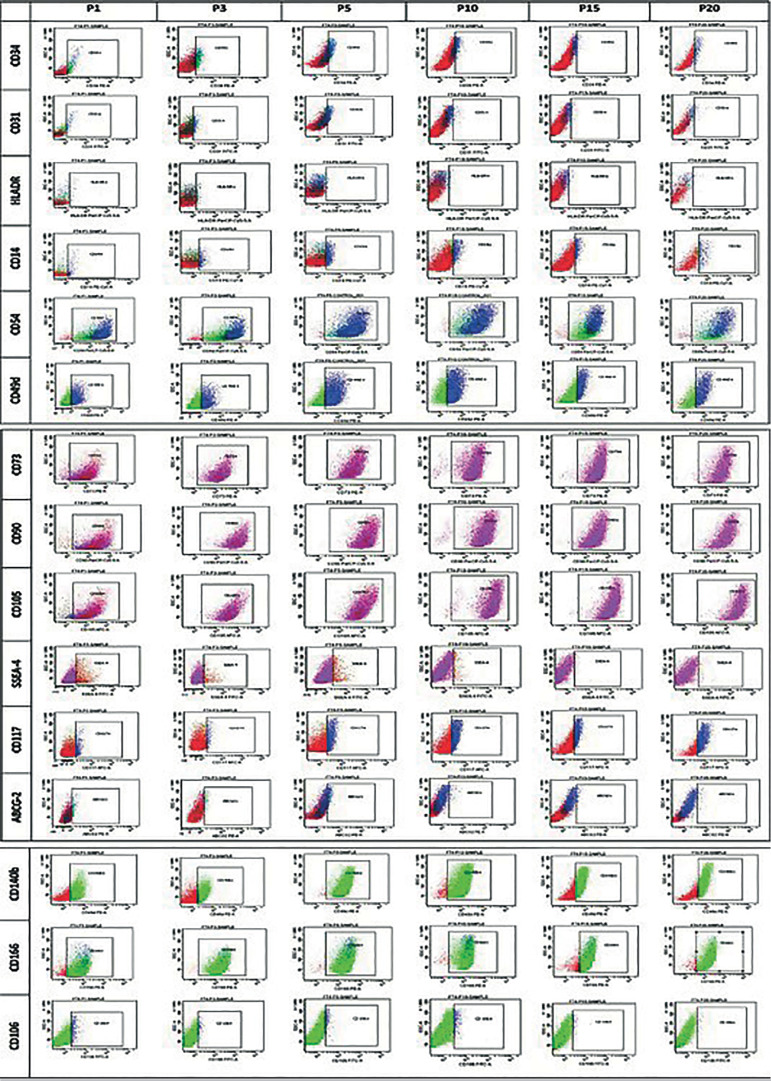
Flow cytometry data

**Figure 2:**
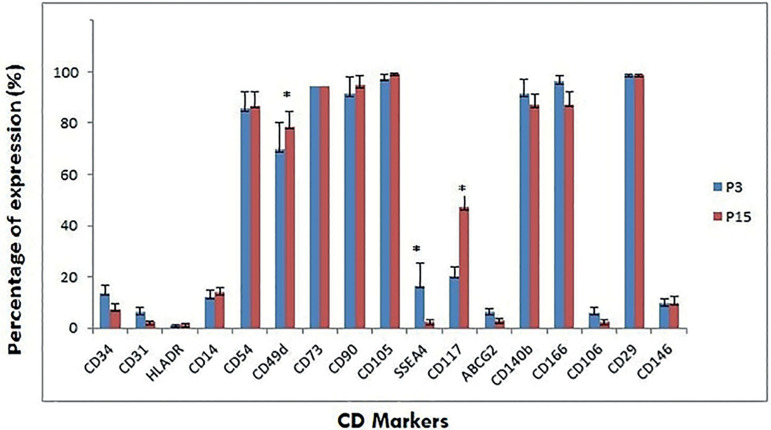
Comparative immunophenotyping at early and late passages. Surface markers expressed as mean±SEM.

The markers used for characterization and their respective fluorochromes are shown in Table 1. Adherent mesenchymal stem cells from fallopian tube were characterized for various markers at different passages such as P1, P3, P5, P10, P15 (Graph 1). The expression profiles in the form of mean±SEM at early (P3) and late (P15) passages were recorded (Fig. 2). The expression profiles of MSCs were similar under extensive culturing; some of the other markers, such as CD34, CD31, SSEA4, ABCG2, and CD146, had a reduced expression in P15 as compared to P3. However, significant variations in marker expressions were found in CD49d, SSEA4, and CD117.

### Proliferative analysis

**Figure 3:**
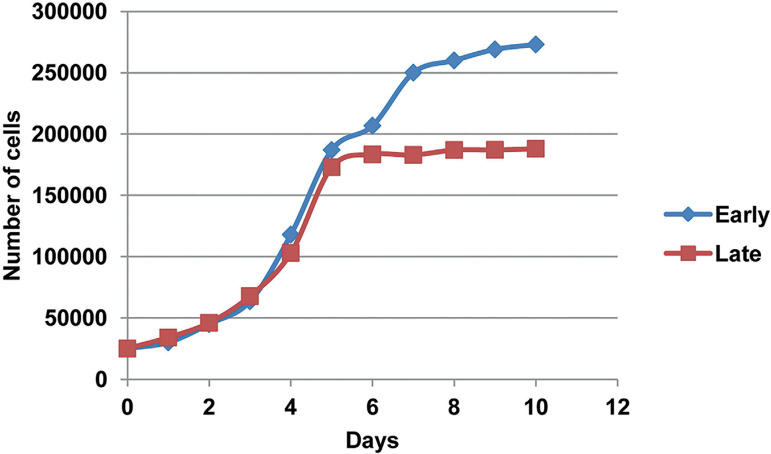
Growth curve analysis. Growth curve analysis of FT-MSCs.

**Figure 4:**
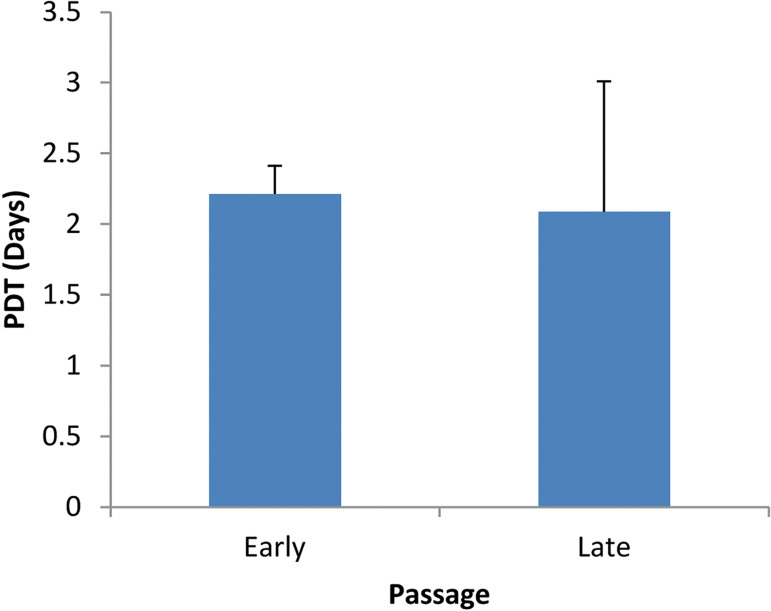
Population doubling time. PDT of FT-MMSCs at early and late passage.

The proliferative capacity and viability of the cultured FT-MSCs were assessed using growth curve (Fig. 3) and PDT analysis (Fig. 4). The proliferation rate of FT-MSCs was lower in the later passage as compared to the early passage condition.

### Differentiation Potential

**Figure 5:**
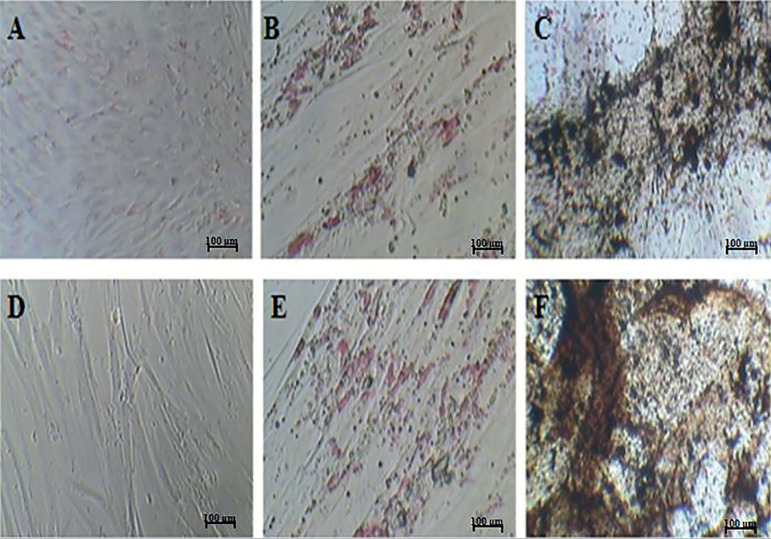
Differentiation study. A,F. Undifferentiated cell; B,E. Adipocyte differentiation (P3); C,D. Osteoblast differentiation at early and late passage, respectively.

The differentiated cells were demonstrated by staining techniques. FT-MSCs showed a higher differentiation pattern overall, unlike the proliferative assay (Fig. 5).

### Karyotyping

**Figure 6:**
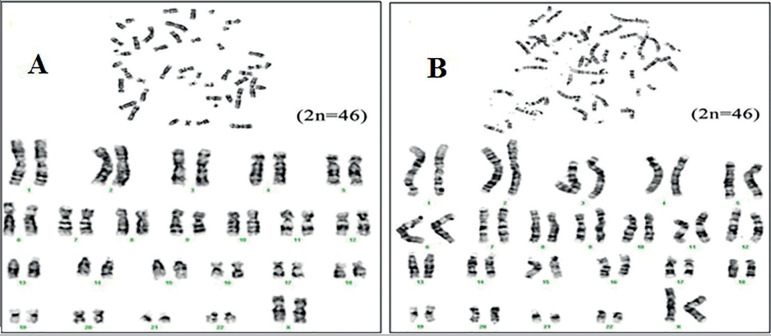
Karyotype ideograms. Karyotype ideograms of FT-MSCs at a. early passage b. late passage.

To evaluate numerical and structural chromosomal ploidy, if any, in view of long-term culture of MSC derived from FT-MSCs, the cells at both early (P3) and late passages (P15) were subjected to karyotyping and GTG banding. GTG banding results of early and late passages of karyotyping were normal (Fig. 6), indicating the retention of diploid status.

## DISCUSSION

The discovery of stem cells led to investigations around potential sources of MSCs procured with minimally invasive techniques and limited ethical qualms (Hematti *et al*., 2013). The majority of stem cell applications in regenerative medicine involve stem cells isolated from bone marrow. However, the isolation of stem cells from BM is highly invasive and does not result in good yield (Faustini *et al*., 2010). Several sources of stem cells have been demonstrated to yield viable stem cells that retain their stemness characteristics. The isolation of MSCs from the fallopian tube (FT-MSCs) was first demonstrated in 2009 (Jazedje *et al*., 2009). These cells were obtained following surgical procedures that involved fallopian tube removal. Despite the extensive knowledge gathered about fallopian tubes, the presence of stem cells in their mucosa is relatively unexplored. Both FT-MSCs and FT-MMSCs have been described as potent sources of autologous reproductive tract injury (Jazedje *et al*., 2009; 2012; Wang *et al*., 2015). The proliferative and secretory properties of FT-MMSCs have also been demonstrated to be superior to those of MSCs. Moreover, their isolation is possible via non-invasive methods, which enables easy isolation of stem cells with limited ethical qualms. Despite these advantages, the ability of FT-MMSCs to retain their stemness under long-term culture conditions needs to be assessed in order to ascertain their therapeutic potential in regenerative medicine.

Our study found that FT-MMSCs possess wide marker characteristics throughout passages such as P3, P5, P10 and P15 as compared to stem cells conventionally procured from bone marrow (Chan *et al*., 2004; Gargett *et al*., 2008; Gargett & Masuda, 2010; Dimitrov *et al*., 2008). Despite similarities in their marker characteristics, a few markers such as CD49d, CD117 and SSEA4 showed a significant difference in their expression pattern at P15. Besides, evidential change in morphology at P15 coupled with reduced growth potential was demonstrated. Morphologically, the cells changed their shape as demonstrated in Figure 1, became non-adherent, and lost their ability to grow beyond P15. However, no karyotype changes were noted at P15. The major risks associated with the use of MSCs are malignant transformation, GvHD, and aging of the in vitro differentiated stem cell population (Tong *et al*., 2021). In order to prevent the risks associated with stem cells in regenerative medicine, the definition of their attributes and efficacy up to specific passages in vitro prior to administration is required. In addition, the differentiation potential, expression of surface markers, and chromosomal ploidy also need to be assessed to ensure they can be used. Thus, overall, due to changes in morphology, non-adherent and reduced growth potential beyond P15, it was postulated that FT-MMSCs could not outweigh the existing potent sources of MSCs such as adipose tissue and bone marrow (Dhanasekaran *et al*., 2012; 2013) in regenerative medicine applications.

Nevertheless, FT-MMSCs retain their stemness potential in early passages, and might therefore be used in treating reproductive tract injuries and other tubal infections. The high differentiation potential observed in our study was similar to the potential reported in studies carried out by Wang *et al*. (2015). Therefore, this study might provide input for researchers working on the potential uses of these stem cells in treating bone diseases, especially osteoporosis, a common condition in postmenopausal women. Previous findings corroborate this application (Jazedje *et al*., 2012; Wang *et al*., 2015) and highlight the use of FT-MSCs and FT-MMSCs in bone-related diseases.

Another interesting avenue that can be explored is the high levels of expression of markers of pericytes in the endometrium (Indumathi *et al*., 2013; Kaingade *et al*., 2017) and lower expression levels in FT-MMSCs, despite their common origins, in studies designed to probe into the roles of stem cells and pericytes in fallopian tubes with normal physiology and disease. This study also opens the door to hypotheses around the potential role dysfunctional FT-MSCs and FT-MMSCs have in tubal infections or tubal hyperplasia and injury.

**Figure 7:**
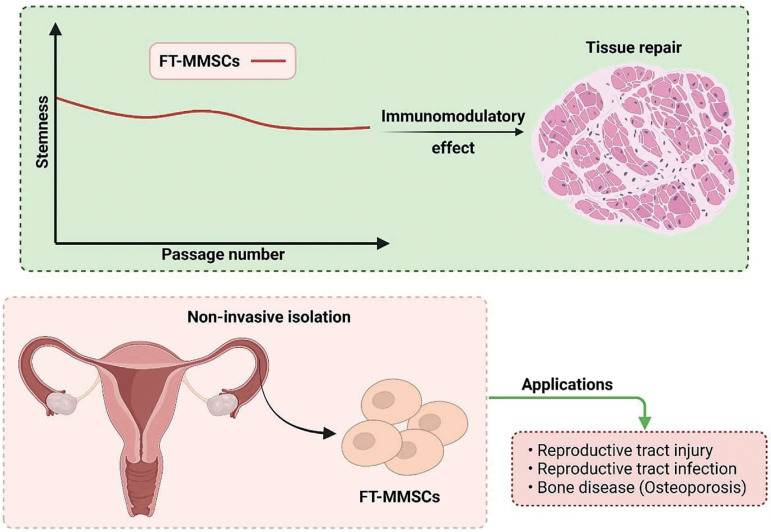
Graphical summary of the study and potential applications The key findings of the study: alteration of stemness characteristics of fallopian tube mucosal mesenchymal stem cells (FT-MMSCs), immunomodulatory properties, and role in tissue repair have been graphically summarized in the figure. The potential applications of FT-MMSCs have also been mentioned.

Further studies need to be conducted to determine the effect of external growth factors on the stemness of FT-MMSCs. Also, the effects of FT-MMSCs on immunomodulation are of paramount importance in regenerative medicine and tissue injury applications. Overall, we recommend the use of FT-MMSCs in stem cell applications that require a high differentiation potential. Since the cells were able to maintain stemness in early passages, they might be utilized in wound healing of reproductive tract injuries and in the treatment of tubal infections by autologous transplantation (Fig. 7). The mucosa might serve as an ideal source for the isolation of mesenchymal stem cells owing to minimal invasiveness and rapid proliferation.

## CONCLUSIONS

Since the fallopian tubes play a major role in the reproductive phases of women, understanding the stem cells procured from this source becomes imperative. Thus, this study was carried out to understand whether FT-MMSCs were able to retain stemness characteristics under long-term culture. Although they did not retain their characteristics as stem cells from other potent sources do, FT-MMSCs might be an alternative element in the treatment of reproductive tract injuries and other tubal infections, although further extensive research on the subject is needed. Besides, as reported earlier, the functionality of FT-MMSCs in fertility and sterility and other pathological reproductive complications is a worthwhile investigation for the betterment of womankind, which might lead more successful reproductive outcomes.

## References

[r1] Chan RW, Schwab KE, Gargett CE (2004). Clonogenicity of human endometrial epithelial and stromal cells. Biol Reprod.

[r2] Deda H, Inci MC, Kürekçi AE, Kayihan K, Ozgün E, Ustünsoy GE, Kocabay S (2008). Treatment of chronic spinal cord injured patients with autologous bone marrow-derived hematopoietic stem cell transplantation: 1-year follow-up. Cytotherapy.

[r3] Dhanasekaran M, Indumathi S, Rashmi M, Rajkumar JS, Sudarsanam D (2012). Unravelling the retention of proliferation and differentiation potency in extensive culture of human subcutaneous fat-derived mesenchymal stem cells in different media. Cell Prolif.

[r4] Dhanasekaran M, Indumathi S, Lissa RP, Harikrishnan R, Rajkumar JS, Sudarsanam D (2013). A comprehensive study on optimization of proliferation and differentiation potency of bone marrow derived mesenchymal stem cells under prolonged culture condition. Cytotechnology.

[r5] Djahanbakhch O, Saridogan E, Kervancioglu ME, Mahmood T, Li L, Grudzinskas JG (1999). Secretory function of the Fallopian tube epithelial cells in vitro: A review. Placenta.

[r6] Dimitrov R, Timeva T, Kyurkchiev D, Stamenova M, Shterev A, Kostova P, Zlatkov V, Kehayov I, Kyurkchiev S (2008). Characterization of clonogenic stromal cells isolated from human endometrium. Reproduction.

[r7] Faustini M, Bucco M, Chlapanidas T, Lucconi G, Marazzi M, Tosca MC, Gaetani P, Klinger M, Villani S, Ferretti VV, Vigo D, Torre ML (2010). Nonexpanded mesenchymal stem cells for regenerative medicine: yield in stromal vascular fraction from adipose tissues. Tissue Eng Part C Methods.

[r8] Gargett CE, Chan RW, Schwab KE (2007). Endometrial stem cells. Curr Opin Obstet Gynecol.

[r9] Gargett CE, Chan RW, Schwab KE (2008). Hormone and growth factor signaling in endometrial renewal: role of stem/progenitor cells. Mol Cell Endocrinol.

[r10] Gargett CE, Masuda H (2010). Adult stem cells in the endometrium. Mol Hum Reprod.

[r11] Gargett CE, Schwab KE, Deane JA (2016). Endometrial stem/progenitor cells: the first 10 years. Hum Reprod Update.

[r12] Ghobadi F, Mehrabani D, Mehrabani G (2015). Regenerative potential of endometrial stem cells: a mini review. World J Plast Surg.

[r13] Hematti P, Kim J, Stein AP, Kaufman D (2013). Potential role of mesenchymal stromal cells in pancreatic islet transplantation. Transplant Rev (Orlando).

[r14] Henriksen T, Tanbo T, Abyholm T, Oppedal BR, Claussen OP, Hovig T (1990). Epithelial cells from human fallopian tube in culture. Hum Reprod.

[r15] Indumathi S, Harikrishnan R, Rajkumar JS, Sudarsanam D, Dhanasekaran M (2013). Prospective biomarkers of stem cells of human endometrium and fallopian tube compared with bone marrow. Cell Tissue Res.

[r16] Jazedje T, Perin PM, Czeresnia CE, Maluf M, Halpern S, Secco M, Bueno DF, Vieira NM, Zucconi E, Zatz M (2009). Human fallopian tube: a new source of multipotent adult mesenchymal stem cells discarded in surgical procedures. J Transl Med.

[r17] Jazedje T, Bueno DF, Almada BV, Caetano H, Czeresnia CE, Perin PM, Halpern S, Maluf M, Evangelista LP, Nisenbaum MG, Martins MT, Passos-Bueno MR, Zatz M (2012). Human fallopian tube mesenchymal stromal cells enhance bone regeneration in a xenotransplanted model. Stem Cell Rev Rep.

[r18] Kaingade P, Nikam A, Kulkarni S, Somasundaram I (2017). Marker profiles of human endometrial stem cells at various passages cultured in-vitro. J Stem Cell Res Ther.

[r19] Karst AM, Drapkin R (2012). Primary culture and immortalization of human fallopian tube secretory epithelial cells. Nat Protoc.

[r20] Li S, Winuthayanon W (2017). Oviduct: roles in fertilization and early embryo development. J Endocrinol.

[r21] Locke M, Windsor J, Dunbar PR (2009). Human adipose-derived stem cells: isolation, characterization and applications in surgery. ANZ J Surg.

[r22] Lyons RA, Saridogan E, Djahanbakhch O (2006). The reproductive significance of human Fallopian tube cilia. Hum Reprod Update.

[r23] Parker AM, Katz AJ (2006). Adipose-derived stem cells for the regeneration of damaged tissues. Expert Opin Biol Ther.

[r24] Pittenger MF, Mackay AM, Beck SC, Jaiswal RK, Douglas R, Mosca JD, Moorman MA, Simonetti DW, Craig S, Marshak DR (1999). Multilineage potential of adult human mesenchymal stem cells. Science.

[r25] Pountos I, Corscadden D, Emery P, Giannoudis PV (2007). Mesenchymal stem cell tissue engineering: techniques for isolation, expansion and application. Injury.

[r26] Prasad K, Mohanty S, Bhatia R, Srivastava MV, Garg A, Srivastava A, Goyal V, Tripathi M, Kumar A, Bal C, Vij A, Mishra NK (2012). Autologous intravenous bone marrow mononuclear cell therapy for patients with subacute ischaemic stroke: a pilot study. Indian J Med Res.

[r27] Rebelatto CK, Aguiar AM, Moretão MP, Senegaglia AC, Hansen P, Barchiki F, Oliveira J, Martins J, Kuligovski C, Mansur F, Christofis A, Amaral VF, Brofman PS, Goldenberg S, Nakao LS, Correa A (2008). Dissimilar differentiation of mesenchymal stem cells from bone marrow, umbilical cord blood, and adipose tissue. Exp Biol Med (Maywood).

[r28] Simari RD, Pepine CJ, Traverse JH, Henry TD, Bolli R, Spoon DB, Yeh E, Hare JM, Schulman IH, Anderson RD, Lambert C, Sayre SL, Taylor DA, Ebert RF, Moyé LA (2014). Bone marrow mononuclear cell therapy for acute myocardial infarction: a perspective from the cardiovascular cell therapy research network. Circ Res.

[r29] Snegovskikh V, Mutlu L, Massasa E, Taylor HS (2014). Identification of putative fallopian tube stem cells. Reprod Sci.

[r30] Stolzing A, Jones E, McGonagle D, Scutt A (2008). Age-related changes in human bone marrow-derived mesenchymal stem cells: consequences for cell therapies. Mech Ageing Dev.

[r31] Tong Y, Zuo J, Yue D (2021). Application Prospects of Mesenchymal Stem Cell Therapy for Bronchopulmonary Dysplasia and the Challenges Encountered. Biomed Res Int.

[r32] Verdi J, Tan A, Shoae-Hassani A, Seifalian AM (2014). Endometrial stem cells in regenerative medicine. J Biol Eng.

[r33] Wang J, Zhao Y, Wu X, Yin S, Chuai Y, Wang A (2015). The utility of human fallopian tube mucosa as a novel source of multipotent stem cells for the treatment of autologous reproductive tract injury. Stem Cell Res Ther.

[r34] Zakrzewski W, Dobrzyński M, Szymonowicz M, Rybak Z. (2019). Stem cells: past, present, and future. Stem Cell Res Ther.

[r35] Zhu Y, Liu T, Song K, Fan X, Ma X, Cui Z (2008). Adipose-derived stem cell: a better stem cell than BMSC. Cell Biochem Funct.

